# Poncet's Disease in the Preclinical Phase of Rheumatoid Arthritis

**DOI:** 10.1155/2018/3571247

**Published:** 2018-05-07

**Authors:** Myat Tun Lin Nyo, Mahmood M. T. M. Ally, Elsa Magreta Van Duuren, Regan Arendse

**Affiliations:** ^1^Department of Medicine, Division of Rheumatology, Sefako Makgatho Health Sciences University, Pretoria, South Africa; ^2^Department of Internal Medicine, Division of Rheumatology, University of Pretoria, Pretoria, South Africa; ^3^Department of Medicine, Division of Rheumatology, Sefako Makgatho Health Sciences University, Pretoria, South Africa; ^4^Division of Rheumatology, Community Rheumatology Care, University of Saskatchewan, 301-39 23rd St. East, Saskatoon, SK, Canada S7K 0H6

## Abstract

We report on a patient with seropositive polyarthritis retrospectively diagnosed as Poncet's disease in the preclinical phase of seropositive rheumatoid arthritis. Our patient developed rheumatoid arthritis more than 2 years after being successfully treated for pulmonary tuberculosis and an initial inflammatory polyarthritis consistent with the diagnosis of Poncet's disease. This case illustrates the importance of recognizing Poncet's disease in a patient presenting with polyarthritis in order to avoid inappropriate long-term disease modifying antirheumatic treatment. It also illustrates the need for adequate follow-up of patients with Poncet's disease after treatment with antituberculosis treatment so that progression to a primary inflammatory arthritis such as rheumatoid arthritis may be identified timeously. Although seropositivity for rheumatoid arthritis has been reported in Poncet's disease as well as in tuberculosis, it is rather uncommon, and long-term follow-up of patients with Poncet's disease is essential particularly if they have positive serological tests for rheumatoid arthritis. In this case report, we describe the first reported case of Poncet's disease in the preclinical phase of rheumatoid arthritis and review the literature related to this rare disease presentation.

## 1. Introduction

Poncet's disease (PD), also known as tuberculosis-associated arthritis, is a nonerosive inflammatory arthritis that may follow a tuberculosis infection without direct mycobacterial presence in the involved joints. PD can mimic rheumatoid arthritis (RA) both clinically and serologically. Serological tests such as rheumatoid factor (RF) and anticitrullinated peptide antibody (ACPA) may be positive in patients with TB without documented clinical evidence of rheumatoid arthritis. In patients with TB without RA, positive ACPA is reported in up to 37% and positive IgM RF in up to 62%. While it is important to recognize PD in a patient presenting with polyarthritis in order to avoid inappropriate long-term disease-modifying antirheumatic treatment, it is also equally important to adequately follow up a patient with PD so that the evolution to a primary inflammatory arthritis such as RA does not go undetected.

## 2. Case Report

In December 2013, a 64-year-old African female was referred to the rheumatology clinic at our hospital with suspected RA. She reported painful hands and knees associated with early morning stiffness of two to three hours for the duration of one year. On further questioning, she reported that she had been diagnosed with pulmonary tuberculosis (TB) on the basis of positive sputum testing eight months prior to the time. The Gene Xpert test on her sputum had detected Mycobacterium tuberculosis complex, which was sensitive to rifampicin. She had subsequently been started on antituberculosis (TB) chemotherapy, which included rifampicin, isoniazid, ethambutol, and pyrazinamide. However, she had taken the treatment for only three months reportedly due to intolerance of the chemotherapy.

Rheumatological examination at that visit revealed a clinical picture suggestive of RA. She had seven swollen joints all of which were metacarpophalangeal joints and seven tender joints including the wrists, knees, and metacarpophalangeal joints. Her patient global assessment was seven, and physician global assessment was four. This indicated high disease activity with a calculated clinical disease activity index (CDAI) of 25. Her chest radiograph showed features suggestive of active tuberculosis in the right upper lobe. Periarticular osteoporosis suggestive of an early inflammatory arthritis was identified on plain radiographs of hands and feet. There were no erosions or joint space narrowing identified on the latter. Blood investigations revealed positive serological tests for RA, with a rheumatoid factor (RF) of 69 international units per millilitre (normal value < 15) and an anti-citrullinated peptide antibody (ACPA) of 53 units per millilitre (normal value < 20). Inflammatory markers were also elevated which could be either due to active arthritis or as a result of active pulmonary tuberculosis.

On the basis of chronic symmetrical polyarthritis involving predominantly small joints, plain radiographs suggestive of an early inflammatory arthritis, and supportive serological features, we diagnosed her arthritic condition as RA. Recognizing that she had not completed her anti-TB chemotherapy and she still had clinicoradiological features suggestive of active pulmonary TB, the original anti-TB chemotherapy was restarted and she was referred to the local clinic to continue the treatment for six months. As for RA, we decided not to initiate methotrexate until anti-TB chemotherapy was completed. We opted instead for a combination therapy of chloroquine and low-dose prednisone for the arthritis. In the follow-up clinic, a month later, we added sulfasalazine as her disease activity was persistently high. She subsequently defaulted the RA treatment. She however continued going to the local TB clinic to complete anti-TB chemotherapy.

When she finally returned to the rheumatology clinic after completion of six months of anti-TB therapy, we found that she had no residual symptoms and signs of RA without any specific therapy for at least 4 months. We felt that a complete resolution of arthritis would be very unlikely under anti-TB chemotherapy without adequate DMARD therapy if the patient had rheumatoid arthritis. This led to a retrospective diagnosis of PD, which is a condition known to completely resolve without joint damage on anti-TB chemotherapy.

**Figure 1:**
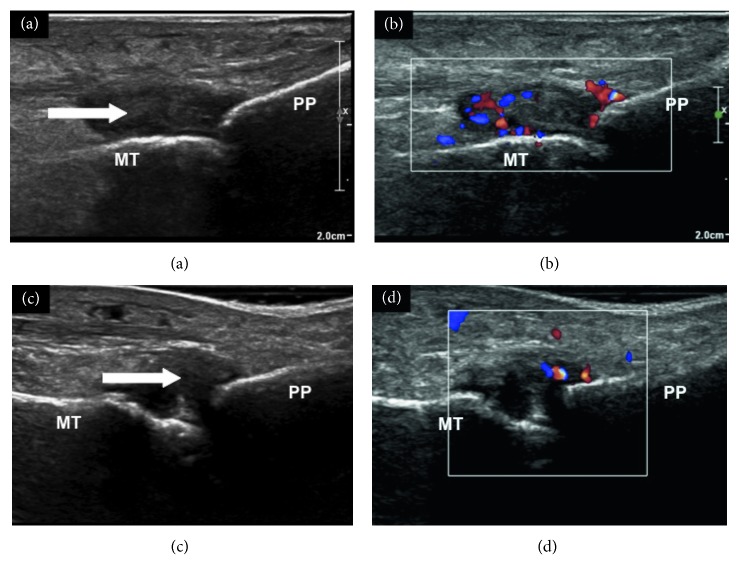
Musculoskeletal ultrasound images showing synovitis of right second and third metatarsophalangeal (MTP) joints. (a) Synovial hypertrophy of right second MTP joint (arrow); (b) increased Doppler signal indicating active inflammation; (c) synovial hypertrophy of right third MTP joint (arrow); (d) increased Doppler signal indicating active inflammation. MT: metatarsal; PP: proximal phalange.

In the subsequent 2-year follow-up without any form of pharmacological treatment, there was no relapse of the arthritis despite the serological tests for RA remaining positive, and she was discharged from the clinic. However, she presented again in January 2017 with a painful right foot, and she had been limping for a few months due to the pain. Clinical examination revealed swelling of the second and third metatarsophalangeal joints and tenderness of all of the right metatarsophalangeal joints. Repeated plain radiographs of the hands and feet revealed periarticular osteoporosis without erosions or joint space narrowing. Musculoskeletal ultrasound revealed active synovitis with increased Doppler signal in the right second and third metatarsophalangeal joints (Figure 1). No erosions were detected in the hand joints on ultrasound examination either. Serum RF was 166 international unit per millilitre, and serum ACPA was 100 units per millilitre. Serum C-reactive protein was mildly elevated at 17 milligrams per litre. She had no features to suggest reactivation of TB or reinfection with TB at this time. RA was diagnosed, and she was then started on oral methotrexate, folate, and low-dose prednisone leading to improvement of her symptoms. Written informed consent was obtained from the patient to allow publication of her clinical diagnosis and management.

## 3. Discussion

PD is a nondestructive (nonerosive) inflammatory arthritis that may follow mycobacterial infection elsewhere with no direct infective agent identified in the involved joints. Antonin Poncet first described it in 1897 [1]. The definition of the disease was modified and improved to a more precise one by Bloxham and Addy in 1987 [2]. Multiple case reports of PD have been published globally since the original description of the disease. In 2013, Rueda et al. [3] systemically reviewed 198 case reports and studies concerning PD published to that date. The clinical characteristics of PD were synthesized from this analysis and a case from their academic centre. Based on the analysis of these 198 cases and a case of their own, PD was hypothesized to be an immunologically mediated disease caused by molecular mimicry between TB antigens and host cartilage in genetically predisposed individuals. The joint involvement improved spontaneously weeks after anti-TB therapy was completed without sequelae. Pulmonary TB was found to be the commonest site of TB infection (43.2%). The sites of extrapulmonary TB infection included lymphatic, renal, bone, skin, and other less common sites. Oligoarthritis was the most common rheumatological presentation (40%) followed by polyarthritis (27.6%) and monoarthritis (24.6%). The most frequently involved joints were the ankles (63%) and knees (59%). The wrists, elbows, interphalangeal joints, metacarpophalangeal joints, shoulders, and metatarsophalangeal joints were affected less frequently. Axial involvement has not yet been reported to date. More recently, Sharma et al. [4] reported a case series of 23 patients with PD. Oligoarthritis was found in 13 patients and polyarthritis in the remaining ten with the ankles being the most frequently involved joints. All patients in the series had nonerosive and nondeforming arthritis.

The RF status of PD has frequently been documented in the previously published case reports and series. The majority of reports that have published the RF status of patients with PD have reported this status to be negative [5]. In contrast, the ACPA status is less often reported and was negative in a few case reports that documented its status. There is, however, a single case of PD that has reported high titres of RF and ACPA, which mimicked rheumatoid arthritis [6]. This case did not report the duration of follow-up after resolution of arthritis or whether there was progression to rheumatoid arthritis. In addition, there are reports that the RF and ACPA tests may be positive in patients with TB without documented clinical evidence of rheumatoid arthritis. In patients with TB without RA, positive ACPA is reported in up to 37% and positive IgM RF in up to 62%. However, it is also not transparent in the latter reports that the patients were followed up for a defined period after treatment of the TB to exclude the progression to rheumatoid arthritis [7–12]. Our case is therefore unique in the literature in that once our patient completed TB therapy with resolution of the PD, the patient subsequently progressed over two years from an ACPA and RF positive status to the extent that she fulfilled the 2010 American College of Rheumatology/European League against Rheumatism (ACR/EULAR) classification criteria for rheumatoid arthritis [13, 14].

The preclinical or seropositive arthralgia phase of RA in which serological tests such as RF and ACPA are positive prior to the onset of clinical phenotype is well described. It is generally recognized that a positive ACPA status is a stronger predictor of progression to rheumatoid arthritis than a positive RF status. The predictors for progression from a seropositive RF and ACPA status to clinically evident rheumatoid arthritis include the demonstration of HLA alleles, *HLA-DRB1 ∗ 0401* and *HLA-DRB1 ∗ 0404*, the presence of raised inflammatory markers, the strength of the ACPA titre, and the presence of subclinical synovitis detected by either ultrasound or MRI examination [15]. Furthermore, it has been postulated that 100% of patients will progress to rheumatoid arthritis within five years if they are both ACPA and RF positive regardless of the background incidence of rheumatoid arthritis in the population from which the patient originates [15]. There is also tentative evidence that preemptive treatment of the arthralgia in a patient with positive ACPA status with anti-CD20 therapy rituximab may delay the onset and the severity of RA [16].

Periarticular osteoporosis is a well-described radiologic observation in both Poncet's disease [17] and early rheumatoid arthritis [18]. Periarticular osteoporosis is defined as “*a decrease in radiographic density in the osseous structure surrounding the joints*” and is frequently described at the proximal phalanges and metacarpals in early rheumatoid arthritis [18]. However, interobserver agreement in controlled studies evaluating the radiographic diagnosis of periarticular osteoporosis is moderate at best [18]. As a consequence of the high observational variation in reporting periarticular osteoporosis in patients with early rheumatoid arthritis, this radiographic feature has not been included in the 2010 ACR classification criteria [18]. The observation of the presence of periarticular osteoporosis in our patient's first radiographs did not allow us to distinguish between rheumatoid arthritis and Poncet's disease. We are confident therefore that our patient's initial clinical course was consistent with a diagnosis of Poncet's disease and that there was the subsequent development of rheumatoid arthritis.

Palindromic rheumatism is a condition in which a person with positive RF and ACPA serology may report self-limiting, intermittent joint pain, and swelling [19]. The episodes of joint pain and swelling may be present for days to weeks. The joint inflammation is described to resolve spontaneously or in response to symptomatic treatment. The episodes of joint inflammation may be interspersed with symptom-free periods that may range from weeks to years [20]. Palindromic rheumatism is distinct from early RA in that it does not have the persistent joint symptoms of pain and swelling for more than 6 weeks, which are required to fulfill the 2010 ACR/EULAR classification criteria for RA. We considered palindromic rheumatism in the differential diagnosis of our patients' condition, as there was a two-year interval of spontaneous improvement not attributed to the use of conventional DMARDs but to the treatment of the pulmonary tuberculosis. However, the initial and January 2017 presentations of small joint pain and swelling for periods of more than one year and more than a few months, respectively, are inconsistent with the definition of palindromic rheumatism which requires that the swelling be present for less than six weeks. For this reason, we do not believe that palindromic rheumatism may account for the two distinct periods of the inflammatory arthritis interspersed by the two-year period of improvement in joint symptoms. Rather the initial and January 2017 periods of joint swelling may be attributed to successive diagnoses of Poncet's disease and rheumatoid arthritis, respectively.

We hypothesize that our patient initially developed PD in response to the untreated tuberculosis infection. We base this hypothesis on the temporal association between the presentation of the polyarthritis and the presence of tuberculosis and the subsequent resolution once the tuberculosis infection had been treated. We think it is unlikely but not impossible that the PD may have been a form of early RA transiently unmasked by TB. Rather, we hypothesize that elimination of the mycobacterial antigen with antituberculosis therapy may have reduced the antigen load and minimized subsequent molecular mimicry with resolution of the PD. We have not found evidence that the antituberculosis therapy may have a beneficial effect on the activity of RA if the initial PD was in fact an early flare of RA. Neither do we think that the initial presentation of PD was a form of palindromic rheumatism. The absence of prior episodes and subsequent absence of similar episodes within the two-year follow-up after completing the TB treatment suggest that the diagnosis of PD is distinct from palindromic rheumatism.

We further hypothesize that our patient subsequently evolved to rheumatoid arthritis as a result of the predictive features of elevated inflammatory markers and strongly positive serology. While we place emphasis on the presence of the predictive features, we acknowledge that it is also possible that the mycobacterial infection may have been contributory to the progression to RA. In this regard, there has been interest in the role of mycobacterial 65-kd heat-shock protein (BHSP65) in the pathogenesis of adjuvant-induced arthritis in rodents and RA in humans [21–24]. Despite the convincing data for the relationship between BHSP65 and experimental arthritis in rodents, the causative role of BHSP65 in human RA remains unproven. We suspect that the case reported by Sasaki et al. [6] of PD with positive RF and ACPA serology may have also eventually progressed to RA if monitored for a similar or longer period of time. The absence of TB infection and the absence of predictive features may have limited the progression to RA within the period reported by Sasaki et al. [6].

## 4. Conclusion

We report a unique case of a patient with seropositive polyarthritis retrospectively diagnosed as PD in the preclinical phase of seropositive RA. She developed RA more than 2 years after being successfully treated for pulmonary TB and an initial inflammatory polyarthritis consistent with the diagnosis of PD. While this case illustrates the importance of recognizing PD in a patient presenting with polyarthritis in order to avoid unnecessary long-term disease-modifying antirheumatic treatment, it also illustrates the need for adequate follow-up in patients with PD so that the possibility of RA is not overlooked. The existence of a preclinical phase of RA in which serological tests such as RF and ACPA can be positive for years before the onset of clinical phenotype is well recognized in the literature. Although seropositivity for RA has been reported in PD as well as in TB, it is rather uncommon and long-term follow-up of patients with PD is essential particularly if they have positive serological tests for RA. This is the first description of PD in the preclinical phase of RA.
